# Enhancing Oral Health Diagnostics With Hyperspectral Imaging and Computer Vision: Clinical Dataset Study

**DOI:** 10.2196/76148

**Published:** 2025-09-11

**Authors:** Paul Römer, Jean-Jacques Ponciano, Katharina Kloster, Fabia Siegberg, Bastian Plaß, Shankeeth Vinayahalingam, Bilal Al-Nawas, Peer W Kämmerer, Thomas Klauer, Daniel Thiem

**Affiliations:** 1 Department of Oral and Maxillofacial Surgery, University Medical Center of the Johannes Gutenberg University Mainz, Augustusplatz 2, Mainz, 55131, Germany, 49 1747978980; 2 Institute for Spatial Information and Surveying Technology, University of Applied Sciences, Mainz, Germany; 3 Department of Oral and Maxillofacial Surgery, Radboud University Nijmegen, Nijmegen, The Netherlands

**Keywords:** hyperspectral Imaging, endoscopic HSI, eHSI, oral mucosa perfusion, ischemia, oral health diagnostics, deep learning, oral squamous cell carcinoma, medical imaging dataset, tissue classification, spectral analysis, oral pathology, artificial intelligence, dentistry, non-invasive diagnostics

## Abstract

**Background:**

Diseases of the oral cavity, including oral squamous cell carcinoma, pose major challenges to health care worldwide due to their late diagnosis and complicated differentiation of oral tissues. The combination of endoscopic hyperspectral imaging (HSI) and deep learning (DL) models offers a promising approach to the demand for modern, noninvasive tissue diagnostics. This study presents a large-scale in vivo dataset designed to support DL-based segmentation and classification of healthy oral tissues.

**Objective:**

This study aimed to develop a comprehensive, annotated endoscopic HSI dataset of the oral cavity and to demonstrate automated, reliable differentiation of intraoral tissue structures by integrating endoscopic HSI with advanced machine learning methods.

**Methods:**

A total of 226 participants (166 women [73.5%], 60 men [26.5%], aged 24‐87 years) were examined using an endoscopic HSI system, capturing spectral data in the range of 500 to 1000 nm. Oral structures in red, green, and blue and HSI scans were annotated using RectLabel Pro (by Ryo Kawamura). DeepLabv3 (Google Research) with a ResNet-50 backbone was adapted for endoscopic HSI segmentation. The model was trained for 50 epochs on 70% of the dataset, with 30% for evaluation. Performance metrics (precision, recall, and *F*_1_-score) confirmed its efficacy in distinguishing oral tissue types.

**Results:**

DeepLabv3 (ResNet-101) and U-Net (EfficientNet-B0/ResNet-50) achieved the highest overall *F*_1_-scores of 0.857 and 0.84, respectively, particularly excelling in segmenting the mucosa (0.915), retractor (0.94), tooth (0.90), and palate (0.90). Variability analysis confirmed high spectral diversity across tissue classes, supporting the dataset’s complexity and authenticity for realistic clinical conditions.

**Conclusions:**

The presented dataset addresses a key gap in oral health imaging by developing and validating robust DL algorithms for endoscopic HSI data. It enables accurate classification of oral tissue and paves the way for future applications in individualized noninvasive pathological tissue analysis, early cancer detection, and intraoperative diagnostics of oral diseases.

## Introduction

Oral diseases, including malignant and premalignant lesions, often occur on pre-existing chronic tissue alterations that are difficult to discern through conventional visual examination. This process heavily relies on the clinician’s expertise and subjective interpretation, frequently necessitating additional diagnostic measures. Available methods include incisional and excisional biopsies, brush biopsies, cytological techniques, and optical approaches. Among these, scalpel biopsy remains the gold standard for diagnosing potentially malignant lesions, offering a diagnostic accuracy of up to 88.9% [1]. However, it involves the partial removal of tissue from a suspicious area for histopathological examination, making it invasive, costly, and potentially inadequate for multilocular lesions. Excisional biopsies, while more reliable due to larger sample sizes, bear the risk of incomplete removal of malignancies and overtreatment in cases of benign lesions [2].

In recent years, less invasive methods, such as brush biopsies, tissue autofluorescence, and chemiluminescence (eg, toluidine blue staining) have been introduced as cost-effective alternatives. Meta-analyses report high sensitivities for these techniques, ranging from 30% to 100% for tissue autofluorescence, 77% for toluidine blue staining, and 91%‐100% for brush biopsies [3-5]. However, these methods have not significantly improved the early detection of oral squamous cell carcinoma. The examiner-dependent variability of less invasive methods can, at worst, delay the accurate diagnosis of oral squamous cell carcinoma, one of the most prevalent malignant tumors globally, accounting for 90%‐95% of all malignant oral cavity pathologies [6-8]. Consequently, the surgical scalpel biopsy remains the diagnostic gold standard [9]. Evidence shows that patients undergoing routine clinical evaluations, including visual inspection and digital palpation, achieve significantly higher 5-year survival rates [10,11].

The integration of advanced imaging technologies into clinical diagnostics is revolutionizing oral health care. Among these innovations, hyperspectral imaging (HSI) stands out as a noninvasive, highly sensitive modality that captures detailed spectral information across hundreds of wavelengths beyond the visible light spectrum. By detecting tissue-specific spectral signatures, HSI combines imaging remission spectroscopy with conventional imaging techniques [12]. HSI acquires spatial and spectral information as a 3D hyperspectral cube in a noncontact, noninvasive, and radiation-free manner. This approach provides vast datasets spanning wide wavelength spectra, enabling the immediate extraction of diagnostically relevant information [13-18]. The efficacy of HSI relies on the distinct spectral signatures of tissues, which result from their absorption, reflection, and refraction properties when illuminated. Each tissue type generates a unique light spectrum that HSI systems can capture, offering the potential for detecting pathological changes with high precision and sensitivity [19].

Endoscopic HSI enhances access to the oral cavity and improves illumination, making it particularly valuable in addressing the growing demand for noninvasive diagnostic methods in oral medicine [20-23]. In this context, the combination of endoscopic HSI with deep learning (DL)–based computer vision techniques provides the potential to significantly enhance diagnostic accuracy, allowing for more comprehensive mapping of the oral cavity and accurate distinction between healthy and pathologically altered mucosa. This offers potential opportunities to avoid unnecessary biopsies and to improve patient-centered therapy by determining individual resection margins. The advanced capabilities of endoscopic HSI, while promising, pose challenges in managing its vast and intricate data output. The spectral data acquired by endoscopic HSI, characterized by tissue-specific signatures, surpass the processing abilities of human observers and traditional analytical tools. This complexity necessitates advanced data analysis, where DL plays a pivotal role. Specialized in interpreting multidimensional datasets, DL uses neural networks to process extensive endoscopic HSI data efficiently, identifying subtle patterns and deviations indicative of pathology that might be missed by human assessment or conventional algorithms [24]. The integration of DL into endoscopic HSI enhances the precision, consistency, and speed of data interpretation, significantly accelerating the diagnostic process. This is especially critical in clinical environments, where timely, accurate real-time analysis can profoundly impact patient outcomes [25-28]. However, implementing DL in endoscopic HSI analysis presents challenges, primarily the limited availability of annotated endoscopic HSI datasets reflecting the complexity and diversity of real-world clinical cases. Existing datasets, often derived from controlled laboratory environments, inadequately prepare DL models for the variability of clinical settings, limiting their practical utility. A review by Cui et al [24] highlights this data scarcity as a major barrier to effective DL analysis in medical imaging, emphasizing the need for specialized datasets. In recent years, DL has also shown great promise in various other domains of medical imaging, including tumor classification in magnetic resonance imaging scans [29] and noise reduction in medical images [30-33]. These studies highlight the versatility and effectiveness of neural networks in processing complex medical images. Building on these advances, the present work applies DL and endoscopic HSI to the oral cavity—an area where such integration remains underexplored despite its clinical potential of enhancing current standards to advance precision and efficacy in oral diagnostics.

## Methods

### Study Cohort

**Figure 1.**
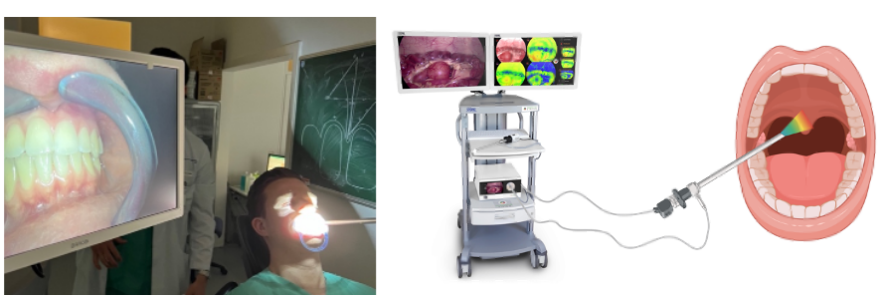
Experimental setup for endoscopic hyperspectral measurements of the oral cavity.

A total of 226 participants, including 166 (73.5%) women and 60 (26.5%) men aged between 24 and 87 years, were included in this prospective, clinical study. Endoscopic HSI data were acquired at the Department of Oral and Maxillofacial Surgery, Facial Plastic Surgery, University Medical Center. The examined participants represented a heterogeneous group of patients from the oral and maxillofacial surgery outpatient clinic. Patients with macroscopic abnormalities of the oral mucosa, premalignant lesions, or tumors were excluded from the study. All participants provided informed consent prior to the procedure and data collection. The individual pictured in Figure 1 has provided permission for their image to be used in this publication. This study was approved by the local ethics committee of Rhineland-Palatinate (registration number 2021‐16158) and was conducted in accordance with the code of ethics of the World Medical Association (Declaration of Helsinki).

### HSI and Patient Data Acquisition

The HSI datasets were acquired using a state-of-the-art endoscopic HSI sensor system (TIVITA Mini^®^ camera system, Diaspective Vision GmbH). This system detects 100 wavelengths in the range of 500‐1000 nm, offering a bandwidth of 5 nm [34]. The device uses the pushbroom principle, enabling chemical component detection based on light absorption and reflection behavior. During operation, light enters the optical system of the spectrometer through the lens, where it is collimated and separated into individual wavelengths using a transmission grating. The separated light then passes through a second optical system before reaching the sensor of the connected complementary metal-oxide-semiconductor camera. The spectrometer directly detects the spatial direction and width of the object being scanned (Y-axis) while the second spatial direction and length of the object (X-axis) are determined through the continuous mechanical movement of the light entry gate within the scanning unit. This process generates a 3D data cube that includes a spectral dimension (λ), capturing complete tissue spectra for each pixel within the wavelength range of 500‐1000 nm [35].

Standardized measurement protocols were followed during image acquisition, maintaining a consistent distance of 7‐10 cm to ensure high-quality and distinct image data (Figure 1). Measurements were performed in an examination room under dimmed lighting conditions to ensure uniformity and comparability. A total of 226 participants aged between 24 and 87 years, including 166 (73.5%) women and 60 men (26.5%), were prospectively recruited at the outpatient department of the clinic of Oral and Maxillofacial Surgery, University Medical Center, where all individuals underwent routine clinical evaluation and inspection. Inclusion was limited to individuals without clinically apparent mucosal lesions, premalignant conditions, or malignancies, ensuring a representative sample of nonpathological oral tissue. Informed consent was obtained from all participants prior to data acquisition. The recruitment strategy was designed to capture a broad range of healthy oral tissue presentations to ensure the generalizability of HSI data across different anatomical sites and patient demographics. For each patient, 5 images were taken, including views of the right and left cheeks, the palate, the back of the tongue, and the closed row of teeth. The data were subsequently pseudonymously archived using camera-specific software tools.

### Red, Green, and Blue–Imaging and Endoscopic HSI Data Files

The Comprehensive Oral Health Hyperspectral Dataset (comprehensive, annotated endoscopic HSI dataset of the oral cavity) primarily comprises RGB (red, green, and blue) and endoscopic HSI data with corresponding annotations. These components in the presented dataset are each important to address the fundamental challenges in DL for semantic segmentation [36], providing robust and effective model training. The RGB component offers standard visual spectrum imagery of the oral cavity, serving as a foundational baseline for comparison with more sophisticated imaging modalities like raw and processed endoscopic HSI data.

### Annotation Process and Verification

Individual annotations of the oral cavity’s anatomical sites were manually performed using an image annotation tool (RectLabel Pro version 2024.06.07, Ryo Kawamura; Tokyo, Japan). To ensure high-quality image data annotation, several key features were implemented. First, detailed annotation guidelines were developed to ensure reproducible results. Comprehensive training sessions and practical exercises for annotators were conducted, followed by feedback rounds to enhance their skills. Each image was annotated by at least 2 independent specialists to minimize subjective errors. Regular quality checks were performed through sample reviews by senior specialists to detect inconsistencies. Continuous feedback was given to annotators, and guidelines were regularly updated based on this feedback. Interannotator analysis of 2 segmentation annotation sets (“original” vs “new”), each assigning a class label (out of 20 possible objects plus background) to every pixel, was carried out additionally for internal quality assurance. Agreement was quantified using metrics that capture both overall and class-specific consistency: (1) Pixel Accuracy, (2) Cohen Kappa (including and excluding background), and (3) Dice Coefficients, which are mathematically equivalent to the *F*_1_-score in the binary case. To ensure a realistic representation of everyday clinical practice, the annotated structures marked for subsequent analysis corresponded to the individual intraoral site and situation, including “clutter (n=752),” “blood (n=3),” “brackets (n=15),” “floor of mouth (n=43),” “gingiva (n=511),” “implant (n=10),” “lip (n=603),” “mucosa (n=745),” “palate (n=539),” “prosthesis (n=65),” “reflection (n=106),” “retractor (n=702),” “suture (n=2),” “telescopic crown (n=28),” “tongue (n=587)” and “tooth (n=681),” with a number of distinct classes varying from a minimal count of 2 for “suture” to a substantial count of 752 for “clutter”.

**Figure 2.**
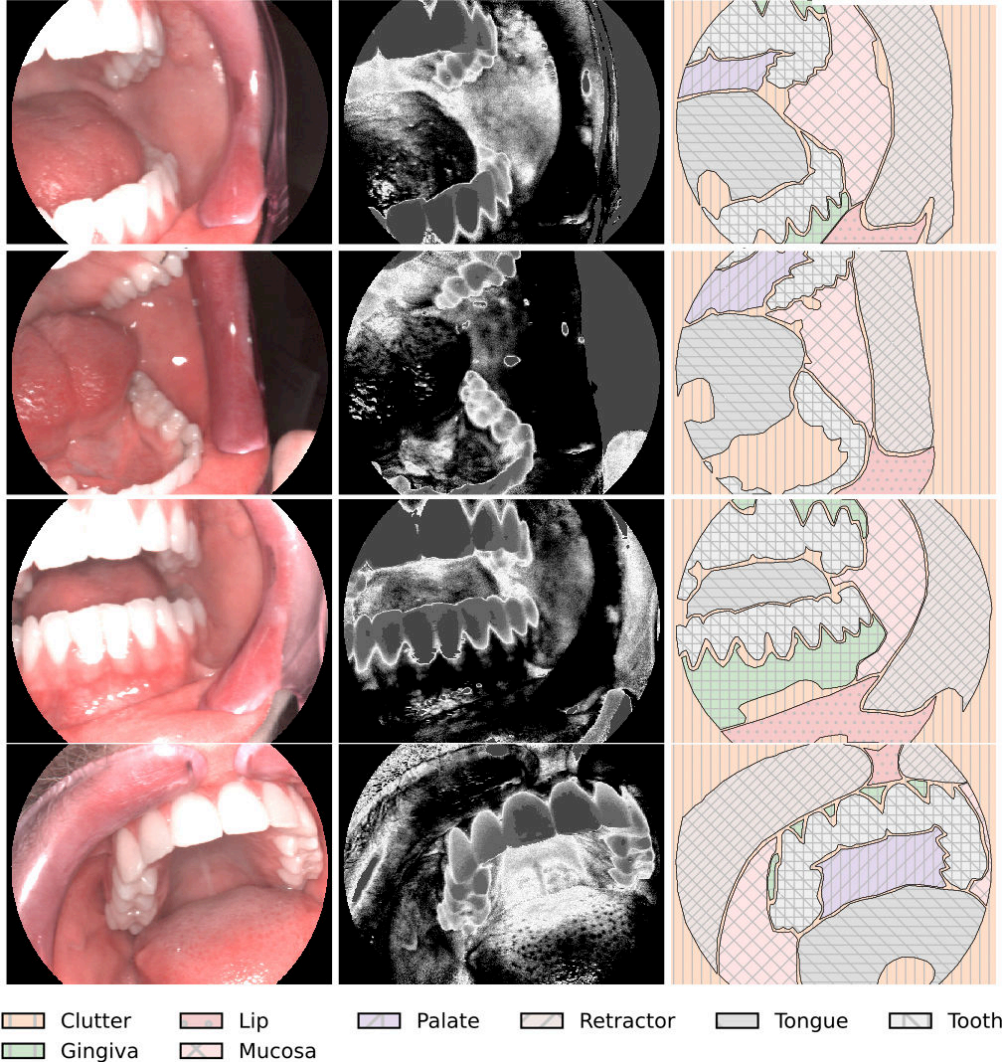
Examples highlighting the detailed annotations, red, green, blue imagery, and features within endoscopic hyperspectral imaging Band 9 (wavelength of 545 nm), which are especially discernible for human eyes.

To minimize diagnostic errors, undersegmentation of anatomical structures was avoided, and experienced clinical experts reviewed each annotation to ensure that all clinically relevant features were thoroughly labeled. This conservative approach occasionally led to small unannotated gaps between each class to prevent the merging of different anatomical sites. The digital annotations in XML format provided a semantic comprehension of the image data, which are essential for the application and training of machine learning and computer vision to differentiate various oral site conditions based on the RGB and endoscopic HSI components (Figure 2).

Preprocessing further involved the conversion of the original hyperspectral data from .DAT format to the NumPy format (.npy) to facilitate interoperability, reproducibility, and ease of use for DL models. Regarding data augmentation, spatial transformations, such as image rotations to enhance model robustness, were used. The addition of noise and blur was intentionally avoided, as these operations could introduce spectral distortions that are not physically plausible in the HSI context.

Given the specific goal of oral structure differentiation, classes with low incidence, such as suture, blood, implant, brackets, telescopic crown, prosthesis, reflection, and floor of mouth were deemed less relevant and excluded from further analysis to prevent skewing the learning process and improve the model’s ability to generalize to more common and diagnostically relevant structures. By focusing on higher-incidence classes, such as palate, retractor, mucosa, tongue, tooth, lip, and gingiva, the training process is optimized, enhancing the model’s efficiency and effectiveness across diverse oral structures. To achieve representative results for the annotation, an additional interannotator analysis and validation was carried out to detect possible person-dependent deviations.

### Dataset Structure

To ensure user-friendly analysis of the hyperspectral dataset and reliable collaboration in the research community, the dataset has been specifically optimized for the Python ecosystem (Python Software Foundation 2023, Python Language Reference, version 3.10) instead of the proprietary software required to load endoscopic HSI data. Regarding scientific computation, Python emerges as the predominant programming language due to its extensive libraries and robust community support. After acknowledging this prevalent preference and recognizing the NPY file format as the most straightforward option for loading data into NumPy, a pivotal library for numerical computations in Python [37], all elements of the comprehensive, annotated endoscopic HSI dataset of the oral cavity (“RGB,” “Annotations,” “HSI_Data_Files”) were archived in the “.npy” format. The NPY format offers several advantages: It is compact and efficient for loading and saving and supports a wide range of data types. This standardization eliminates the complexity and potential incompatibilities associated with proprietary formats. In order to provide high-quality, detailed annotations of the complex intraoral structures, digital annotations were elaborated in XML format. To enhance utility and facilitate integration into researchers’ workflows, a semantic map of the oral cavity was generated directly from the XML datasets for each image. These maps essentially consisted of binary or multiclass masks, delineating the categorization of each pixel based on the detailed information provided in XML. However, the creation of these maps is an automated process that translates the complex structured information of the XML annotations into a simple but comprehensive format that can easily be integrated into machine learning models to differentiate tissue types based on the values of each pixel corresponding to the specific annotated category. The workflow in short: First, eHSI endoscopic HSI data are loaded, and semantic information is extracted from the XML file. This information is then used to generate a semantic map of the oral cavity, where each pixel value corresponds to a specific annotated category, such as different tissue types or anatomical sites. The resulting semantic map provides comprehensive, pixel-wise annotations of the frame, ready for application in segmentation algorithms.

### Statistical Analysis of the Dataset

For an initial assessment of the spectral data, a coefficient of variation (CV) was used as a statistical measure. The CV is a standardized measure for dispersion of a probability distribution or frequency distribution and defines the ratio of the SD to the mean, expressed as percentage. It is particularly useful in the context of HSI, as it allows the comparison of variation between different classes and bands despite the different mean intensities. The formula used for the coefficient of variation is:


$$CV=\left(\frac{\sigma }{\mu }\right)\times 100\%$$

where σ is the SD of the dataset and μ is the mean of the dataset.

### Machine Learning and DL Techniques

This study adapted several state-of-the-art models and their performance assessing semantic segmentation in the context of oral health diagnostics. These models included DeepLabv3 [38-40], as well as fully convolutional network (FCN [41]) and pyramid scene parsing network (PSPNet [42]), each tested with ResNet-50 and ResNet-101 [43], backbones. In addition, PSPNet using VGG16 [44] and U-Net [45] models using both EfficientNet-B0 [46] and ResNet-50 backbones were evaluated. Each model was optimized for processing our dataset, which features challenging anatomical structures, enabling a comprehensive analysis of their suitability for handling the variability inherent in endoscopic HSI data for oral health diagnosis. The models were trained over 50 epochs using 70% of the comprehensive, annotated endoscopic HSI dataset of the oral cavity test set, with the remaining 30% reserved for evaluation. This split allowed for a thorough assessment of the models’ ability to generalize to unseen data. Each model’s performance on endoscopic HSI data was evaluated using Precision, Recall, and the *F*_1_-score, providing a comprehensive measure of their effectiveness in segmenting and classifying the various anatomical structures present in the dataset.

### Ethical Considerations

The study was approved by the local ethics committee of Rhineland-Palatinate (registration number: 2021-16158) and was conducted in accordance with the protocol and in compliance with the moral, ethical, and scientific principles governing clinical research as set out in the Declaration of Helsinki of 1975 as revised in 1983. Informed consent was obtained from all participants involved in the study. This study received approval from the Ethics Committee of the Medical Association of Rhineland-Palatinate (reference number 2021-15858). All procedures adhered to institutional and national ethical standards and were conducted in accordance with the Declaration of Helsinki. Participants were provided comprehensive information regarding the study’s nature, purpose, procedures, data usage, and the potential publication of anonymized images or data related to their participation. The privacy and confidentiality of all participants were rigorously protected. No identifying information, including names or hospital IDs, has been included in the manuscript. Images containing identifiable features were excluded except for Figure 1, which depicts the corresponding author (PR), who has provided written consent for the use of their image in this publication. No financial or material compensation was offered or provided to the participants in this study.

## Results

### Variability of the Dataset

**Figure 3.**
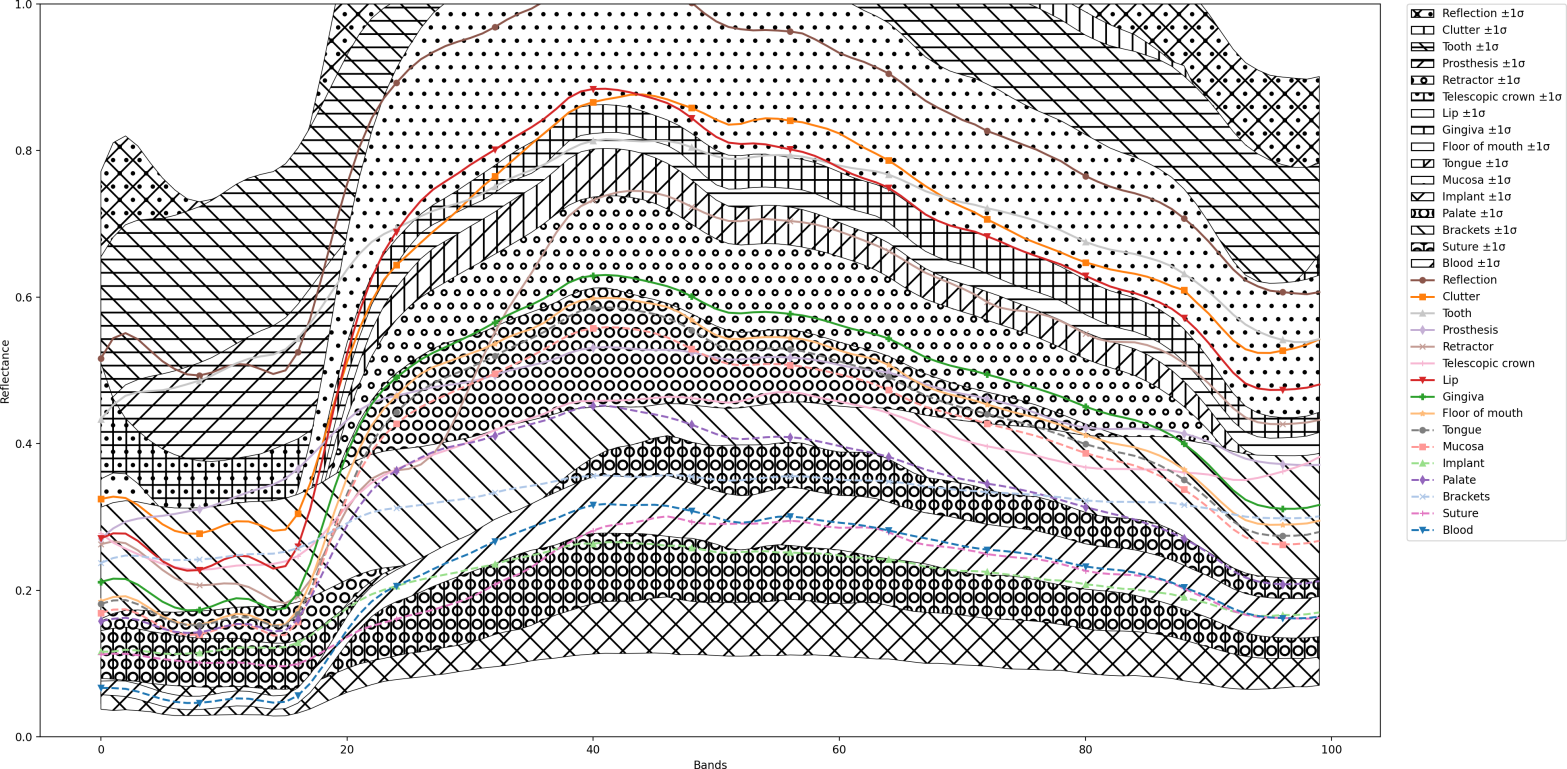
Consolidated diagram of mean values and SD for each class across all measured spectral bands.

A descriptive statistical analysis of the hyperspectral dataset was performed to describe the distribution and variability of the data. Mean values and SD across the spectral bands were analyzed for each class delineated in the hyperspectral images. The objective of the statistical data evaluation was to accurately represent the central tendencies and variabilities of spectral signatures, which are indicative of various anatomical and pathological entities in oral health. Mean values and SDs for all classes across all spectral bands were presented in a consolidated diagram (Figure 3).

**Figure 4.**
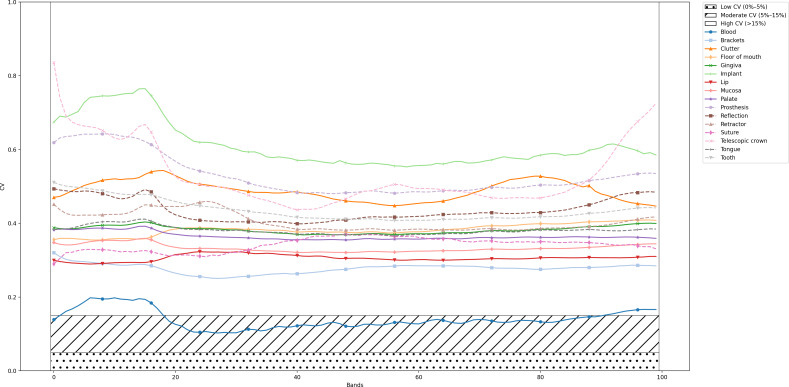
Coefficient of variation over the corresponding bandwidth of various examination classes. CV: coefficient of variation.

The results indicated that the CV for the majority of classes, across most spectral bands, substantially exceeded the commonly recognized threshold of 15%, which is generally considered high. In contrast, a CV below 5% would have denoted homogeneous data, while values between 5% and 15% would have indicated moderate uniformity. The consistently elevated CV values observed across all classes thus underscored significant spectral diversity within each class (Figure 4).

### DL Model Performance

The DeepLabv3 model with the ResNet-50 backbone achieved solid overall performance, with an *F*_1_-score of 0.855, performing especially well on the segmentation of mucosa, retractor, and tooth. However, it exhibited moderate performance on gingiva and lip, with *F*_1_-scores of 0.753 and 0.709, respectively. Switching to the deeper ResNet-101 backbone improved the overall performance slightly, with an *F*_1_-score of 0.857, particularly enhancing the model’s ability to segment clutter and gingiva (Table 1).

Both FCN-ResNet-50 and FCN-ResNet-101 demonstrated robust segmentation performance, achieving overall *F*_1_-scores of 0.862 and 0.861, respectively (Table 2). They excelled in segmenting retractor (*F*_1_-score=0.942 for both), tooth (*F*_1_-score=0.910), palate (*F*_1_-score=0.890), and mucosa (*F*_1_-score≥0.912). However, lip and gingiva posed challenges for both backbones, reflected by lower *F*_1_-scores around 0.72 and 0.77, respectively. Although switching to the deeper ResNet-101 backbone yielded marginal improvements in certain classes, the overall performance remained comparable between the 2 architectures.

PSPNet models showed slightly lower performance, with the ResNet-50 backbone achieving an *F*_1_-score of 0.837 and the VGG16 backbone scoring 0.808. While these models handled classes, such as retractor and tooth, relatively well, they faced difficulties in the segmentation of gingiva and lip, particularly when using VGG16. The lower overall performance of PSPNet VGG16 compared to ResNet-50 reflects the influence of the backbone on the segmentation outcomes (Table 3).

U-Net (EfficientNet-B0) and U-Net (ResNet-50) both demonstrated robust segmentation performance, attaining overall *F*_1_-scores of 0.867 and 0.840, respectively (Table 4). They excelled particularly at segmenting retractor (*F*_1_-score=0.941 vs 0.927), palate (*F*_1_-score=0.909 vs 0.881), and mucosa (*F*_1_-score=0.920 vs 0.901). Tongue and tooth segmentation also performed well, with F1-scores exceeding 0.85 for both models. However, both networks faced challenges in segmenting lip (*F*_1_-score=0.755 vs 0.695) and gingiva (*F*_1_-score=0.751 vs 0.721), indicating opportunities for further refinement in these classes.

**Figure 5.**
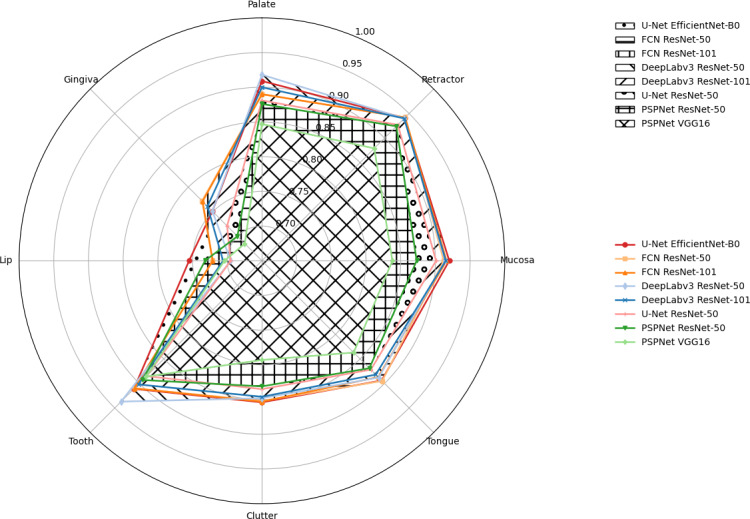
Spider plot illustrating the per-class *F*_1_-score performance of different deep learning models for oral tissue segmentation.

Figure 5 demonstrates that DeepLabv3 (ResNet-101) and U-Net (EfficientNet-B0) outperform other models across most tissue classes, with lower *F*_1_-scores consistently observed for lip and gingiva segmentation.

**Table 1.** Results showing precision, recall, and *F*_1_-score for different classes in a comparison of DeepLabv3 ResNet-50 and DeepLabv3 ResNet-101.

Class	Precision		Recall		*F*_1_-score	
	DeepLabv3 ResNet-50	DeepLabv3 ResNet-101	DeepLabv3 ResNet-50	DeepLabv3 ResNet-101	DeepLabv3 ResNet-50	DeepLabv3 ResNet-101
Overall	0.849	0.851	0.861	0.863	0.855	0.857
Palate	0.886	0.874	0.918	0.929	0.901	0.900
Retractor	0.936	0.942	0.940	0.938	0.938	0.940
Mucosa	0.915	0.914	0.914	0.916	0.914	0.915
Tongue	0.878	0.872	0.888	0.892	0.883	0.882
Clutter	0.840	0.844	0.848	0.849	0.844	0.846
Tooth	0.862	0.871	0.937	0.936	0.898	0.902
Lip	0.723	0.730	0.695	0.683	0.709	0.706
Gingiva	0.756	0.757	0.751	0.765	0.754	0.761

**Table 2.** Results showing precision, recall, and *F*_1_-score for different classes in a comparison of FCN-ResNet-50 and FCN-ResNet-101.

Class	Precision		Recall		*F*_1_-score	
	FCN^a^-ResNet-50	FCN-ResNet-101	FCN^a^-ResNet-50	FCN-ResNet-101	FCN^a^-ResNet-50	FCN-ResNet-101
Overall	0.850	0.850	0.874	0.873	0.862	0.861
Palate	0.859	0.860	0.924	0.923	0.890	0.890
Retractor	0.940	0.935	0.944	0.949	0.942	0.942
Mucosa	0.904	0.907	0.921	0.929	0.912	0.916
Tongue	0.881	0.882	0.909	0.893	0.895	0.887
Clutter	0.864	0.863	0.839	0.843	0.852	0.853
Tooth	0.878	0.876	0.946	0.910	0.910	0.910
Lip	0.699	0.718	0.744	0.723	0.721	0.721
Gingiva	0.776	0.765	0.767	0.777	0.772	0.771

a FCN: fully convolutional network.

**Table 3.** Results showing precision, recall, and *F*_1_-score for different classes in a comparison of PSPNet-ResNet-50^a^ and PSPNet-VGG16.

Class	Precision		Recall		*F*_1_-score	
	PSPNet-ResNet-50	PSPNet-VGG16	PSPNet-ResNet-50	PSPNet-VGG16	PSPNet-ResNet-50	PSPNet-VGG16
Overall	0.849	0.797	0.830	0.822	0.837	0.809
Palate	0.840	0.854	0.915	0.840	0.876	0.847
Retractor	0.929	0.853	0.919	0.906	0.924	0.879
Mucosa	0.906	0.835	0.842	0.841	0.873	0.838
Tongue	0.903	0.794	0.837	0.886	0.869	0.837
Clutter	0.793	0.807	0.873	0.779	0.831	0.793
Tooth	0.877	0.865	0.910	0.907	0.893	0.886
Lip	0.766	0.716	0.701	0.693	0.732	0.704
Gingiva	0.775	0.651	0.639	0.723	0.700	0.685

a PSPNet: pyramid scene parsing network.

**Table 4.** Results showing precision, recall, and *F*_1_-score for different classes in a comparison of U-Net-EfficientNet-B0 and U-Net-Res

Class	Precision		Recall		*F*_1_-score	
	U-Net-EfficientNet-B0	U-Net-ResNet-50	U-Net-EfficientNet-B0	U-Net-ResNet-50	U-Net-EfficientNet-B0	U-Net-ResNet-50
Overall	0.853	0.846	0.882	0.836	0.867	0.840
Palate	0.892	0.857	0.927	0.907	0.909	0.881
Retractor	0.956	0.936	0.927	0.918	0.941	0.927
Mucosa	0.915	0.918	0.924	0.884	0.920	0.901
Tongue	0.893	0.856	0.895	0.889	0.894	0.872
Clutter	0.862	0.811	0.846	0.862	0.854	0.835
Tooth	0.876	0.875	0.949	0.894	0.911	0.885
Lip	0.711	0.734	0.806	0.661	0.755	0.695
Gingiva	0.723	0.780	0.782	0.671	0.751	0.721

### Interannotator Agreement

The analysis of segmentation annotations revealed an overall pixel accuracy of 80.84%, indicating a high level of interannotator agreement. Cohen Kappa, a more robust measure of interannotator agreement, was 77.56% when including the background class, indicating substantial agreement. When the background was excluded, Kappa increased to 82.91%, highlighting even stronger agreement for the segmentation of foreground structures. Dice coefficients per class ranged from near-zero (in classes with minimal or inconsistent labeling) to 93% in well-defined classes, where the overlap between annotations was nearly perfect. Since the Dice Coefficient measures spatial overlap between segmentation masks, high Dice scores (above 90%) indicate excellent agreement, whereas lower values suggest potential ambiguity or underrepresentation of certain structures.

## Discussion

### Principal Findings and Comparison With Previous Works

The objective of this study was to establish a digital map of the oral cavity using endoscopic HSI in conjunction with advanced machine learning techniques and therefore to develop a comprehensive endoscopic HSI dataset. This approach aimed to enable reliable and automated differentiation of various tissue types and objects based on the spectral data acquired from the endoscopic HSI system. Despite the continuous interest in faster, minimally invasive diagnostic techniques, methods, such as brush biopsies and in vivo fluorescence procedures have faced challenges in establishing themselves as reliable alternatives due to their comparatively lower sensitivity and specificity [2,4,5,9]. Modern methods, such as endoscopic HSI, represent a state-of-the-art, innovative approach in the field of automated image and tissue classification. In our preliminary ex vivo studies, we have already demonstrated that HSI can differentiate between various tissue types and states based on specific wavelength patterns [20,47]. However, to effectively differentiate between pathological and healthy tissue conditions in vivo, a substantial dataset of hyperspectral signatures from healthy tissues is essential [47].

The study introduces a comprehensive collection of 1,130,751 endoscopic HSI–cubes of healthy oral mucosa, captured in vivo from various angles, creating a representative digital map that includes relevant tissues and objects of the oral cavity. This dataset forms a representative digital endoscopic HSI map, encompassing relevant tissues and structures of the oral cavity. By establishing a robust reference for healthy tissue, this dataset lays the groundwork for advancing HSI-based diagnostics, particularly in the identification of premalignant and malignant mucosal lesions and the precise definition of tumor resection margins.

The findings revealed elevated CV across most classes, indicating substantial spectral diversity in endoscopic HSI data. This high variability poses challenges for threshold-based classification methods, as intraclass variation can lead to class overlap. However, this diversity also holds valuable information that, when leveraged by advanced computational models, can enhance tissue classification and pathological anomaly detection with high accuracy.

To address this complexity, this study evaluated several state-of-the-art segmentation models, including DeepLabv3, FCN, PSPNet, and U-Net, with different backbones, such as ResNet-50, ResNet-101, VGG16, and EfficientNet-B0. These architectures were chosen for their effectiveness in semantic segmentation, balancing feature extraction capability, computational efficiency, and global context recognition. DeepLabv3 was selected for its ability to handle scale variability and capture fine details while FCN served as a strong baseline due to its foundational role in image segmentation. PSPNet was included for its strong capability in capturing global context, which is crucial for recognizing complex structures in medical imaging. U-Net, widely used in medical imaging, was chosen for its ability to achieve accurate segmentation even with limited data.

The choice of backbones was guided by their specific strengths. ResNet-50 and ResNet-101 were selected for their robust feature extraction, VGG16 for its simplicity and high-resolution detail, and EfficientNet-B0 for its optimized architecture that balances performance and computational efficiency. By incorporating models with varying depths and parameter complexities, this study ensures a comprehensive evaluation of segmentation performance while maintaining a focus on efficient training and inference times. The integration of the DeepLabv3 model with the ResNet-50 as well as with the ResNet-101 backbone offers a robust semantic segmentation approach for endoscopic HSI data interpretation. The primary modification to all the models, including DeepLabv3, FCN, PSPNet, and U-Net (with backbones, such as ResNet-50, ResNet-101, EfficientNet-B0, and VGG16), involved adapting the first convolutional layer to handle the variable number of channels in the dataset. This adjustment was essential to accommodate the multidimensional spectral data of the HSI dataset, as opposed to the standard 3-channel RGB imagery. This modification enables the models to use the unique spectral information in the HSI data cubes beyond the visible spectrum. Despite these adjustments, the deep residual learning architectures of ResNet-50 and ResNet-101 retain their capacity to extract high-level features—an essential aspect of HSI data analysis [43]. These backbones excel at identifying subtle spectral patterns crucial for accurate disease detection and classification. Leveraging atrous convolution and atrous spatial pyramid pooling, the DeepLabv3 model efficiently captures multiscale information [48]. This capability facilitates precise image segmentation, an essential feature for diagnosing oral health conditions with subtle and overlapping visual signatures. Likewise, FCN and PSPNet, with their robust architectures, and U-Net, recognized for its effectiveness in medical imaging, also benefited from the capacity to process and interpret multidimensional spectral data.

The endoscopic HSI procedure implemented in this study achieves results that are comparable to those obtained in preliminary HSI-ex vivo trials conducted by our research group in 2021 [47]. In this study, using a similar approach with a lightweight 6-layer deep neural network containing 10,445 parameters trained over 4000 epochs, tissue samples of fat, muscle, and oral mucosa could be differentiated with an overall class accuracy of over 80%. Similarly, in a study by Ma et al, tissue from various organs, including the kidney, liver, lung, muscle, salivary gland, and spleen, was identified using automated polarized HSI with an accuracy of up to 87% [49]. Furthermore, a deep convolutional neural network established by Poonkuzhali et al [50] could accurately identify brain tissue with an *F*_1_-score precision of 97.3% using HSI in a recent study conducted in 2023. Unlike previous studies that focused on ex vivo tissue samples and organ-specific HSI data [47], the present work introduces the first large-scale in vivo annotated endoscopic HSI dataset of the oral cavity acquired under realistic clinical conditions. While earlier studies demonstrated the technical feasibility of HSI-based tissue differentiation, these approaches often lacked anatomical complexity, semantic annotations, and clinical variability. This study addresses these deficiencies by providing a large annotated in vivo HSI dataset and evaluating multiple DL models especially adapted for HSI data analysis. This provides the basis for clinically applicable segmentation of oral tissues and paves the way for future studies involving pathological lesions.

These findings affirm that HSI, when integrated with appropriate modalities, can serve as a reliable tool for differentiating various tissues in both in vivo and ex vivo setups. In oncological surgery, fast, reliable, and minimally invasive diagnosis of pathological tissue conditions is of paramount importance. Numerous studies in this field have shown that HSI can dependably differentiate between tumor and healthy tissue by analyzing histopathological sections and their spatial-spectral features. Moreover, the combination of HSI and DL has shown superior results compared to the use of RGB images and conventional support-vector-machine approaches [51-56].

In this study, the established DL and neural network approach was able to accurately identify different types of oral mucosa with an overall precision of approximately 91%. DeepLabv3 (ResNet-101) and U-Net (EfficientNet-B0) emerged as the top performers, demonstrating robust segmentation across key anatomical classes. While all models could benefit from further refinement in gingiva and lip segmentation, their consistent accuracy in identifying retractors and teeth underscores the strength of the dataset. While there is no universally accepted threshold for clinical applicability, *F*_1_-scores above 0.85 are generally considered promising in similar biomedical imaging tasks. Scores in this range suggest that the method may already approach a level of accuracy relevant for clinical decision-making, though further validation in real-world settings would be required. Notably, U-Net (EfficientNet-B0) exhibited exceptional segmentation of retractors, mucosa, and teeth, indicating its strong potential for medical image segmentation applications. The results demonstrate the dataset’s suitability for training advanced neural networks, particularly in challenging medical segmentation tasks.

Limitations of our study specifically include the acquisition of a higher amount of data, which depended heavily on the individual patient volume of the clinic conducting the study. This represents a major challenge, particularly when aiming to document various oral mucosal lesions and precancerous mucosal conditions. To address the issue of data scarcity, the raw data generated in this study will be made publicly available. This initiative aims to provide other research groups with the opportunity to use and build upon the dataset for further investigations. Furthermore, while the study cohort was intentionally designed to include a broad cross-section of patients from a university-based outpatient clinic, selection bias cannot be fully excluded. As recruitment was confined to a single university clinic, the cohort may not fully reflect the demographic and clinical variability found in primary care or the general population. Other limitations include variable lighting conditions, motion artifacts, and spectral overlaps, as well as overexposure and underexposure. Overexposure can occur due to various factors, such as the variability in patient anatomy, the movement during image capture, or the fluctuation of lighting conditions in an operating room. While overexposed images are typically regarded as artifacts and excluded from datasets, our approach incorporates them, acknowledging that such occurrences are an inherent part of clinical practice. Understanding that these instances present both challenges and opportunities for advanced image processing techniques, we have carefully indexed these overexposed images for further analysis. This approach enhances the resilience and adaptability of the models trained on this dataset, ensuring they perform effectively in real clinical environments. By incorporating overexposed images, the models are better equipped to handle the full spectrum of data variability, including common environmental factors. This combination highlights the substantial potential of endoscopic HSI data to significantly enhance the precision and reliability in detecting and categorizing healthy and pathological oral conditions. This convergence not only paves the way for significant advancements in diagnostic methodologies but also holds the potential to substantially enhance patient care. External validation is currently planned as part of future work, particularly for mucosa segmentation. We intend to validate our model on an ex vivo dataset that includes mucosa samples captured under different acquisition conditions. At this stage, the focus of this study was the initial validation on the internally collected dataset. Corresponding follow-up studies are currently in preparation and will include HSI of pathological tissue conditions, such as oral mucosal lesions and neoplastic changes, to assess the transferability and diagnostic robustness of the proposed models in clinically relevant scenarios. By enabling earlier detection and more precise characterization of oral health conditions, this approach facilitates the development of more effective treatment strategies, ultimately leading to improved patient outcomes and a higher standard of care.

### Conclusion

This study presents the first large-scale in vivo annotated dataset of the oral cavity using endoscopic HSI under realistic clinical conditions. By combining hyperspectral datasets and DL-based segmentation, it was possible to demonstrate the feasibility of automated, noninvasive tissue classification across important anatomical intraoral structures. Comparative analysis reveals DeepLabv3 and U-Net as robust architectures for oral tissue classification. The comprehensive dataset consequently provides a sufficient foundation for future work on pathological tissue detection, intraoperative margin assessment in oncology, and early, individualized diagnostics in oral medicine.

## Abbreviations

CV: coefficient of variation
DL: deep learning
FCN: fully convolutional network
HSI: hyperspectral imaging
PSPNet: pyramid scene parsing network
